# The Fanconi Anemia Pathway Protects Genome Integrity from R-loops

**DOI:** 10.1371/journal.pgen.1005674

**Published:** 2015-11-19

**Authors:** María L. García-Rubio, Carmen Pérez-Calero, Sonia I. Barroso, Emanuela Tumini, Emilia Herrera-Moyano, Iván V. Rosado, Andrés Aguilera

**Affiliations:** 1 Centro Andaluz de Biología Molecular y Medicina Regenerativa CABIMER, Universidad de Sevilla, Seville, Spain; 2 Instituto de Biomedicina de Sevilla-Hospital Virgen del Rocío, Seville, Spain; The University of North Carolina at Chapel Hill, UNITED STATES

## Abstract

Co-transcriptional RNA-DNA hybrids (R loops) cause genome instability. To prevent harmful R loop accumulation, cells have evolved specific eukaryotic factors, one being the BRCA2 double-strand break repair protein. As BRCA2 also protects stalled replication forks and is the FANCD1 member of the Fanconi Anemia (FA) pathway, we investigated the FA role in R loop-dependent genome instability. Using human and murine cells defective in FANCD2 or FANCA and primary bone marrow cells from FANCD2 deficient mice, we show that the FA pathway removes R loops, and that many DNA breaks accumulated in FA cells are R loop-dependent. Importantly, FANCD2 foci in untreated and MMC-treated cells are largely R loop dependent, suggesting that the FA functions at R loop-containing sites. We conclude that co-transcriptional R loops and R loop-mediated DNA damage greatly contribute to genome instability and that one major function of the FA pathway is to protect cells from R loops.

## Author Summary

R loops are co-transcriptional RNA-DNA hybrids that can have a physiological role in transcription and replication, but also may be a major threat to genome stability. To avoid the deleterious effects of R loops, specific factors prevent their formation or facilitate their removal. The double-strand break repair factor BRCA2 is among those that prevent R-loop accumulation. As BRCA2 also protects stalled replication forks and is the FANCD1 member of the Fanconi Anemia (FA) pathway, we studied the role of this pathway in preventing R loop accumulation and R loop-dependent genome instability. Using human and murine cells defective in FANCD2 or FANCA and primary bone marrow cells derived from FANCD2 deficient mice, we show that the FA pathway removes R loops and that many DNA breaks accumulated in FA cells are R loop-dependent. Importantly, FANCD2 foci accumulation is largely R loop-dependent, suggesting that the FA functions at R loop-containing sites. The FA pathway is primarily known as a DNA interstrand crosslinks (ICLs) repair pathway. Our findings reveal a novel function of the FA pathway in preventing R loop-mediated DNA damage, providing new clues to understand the relevance of R-loops as a natural source of genome instability and the way they are processed.

## Introduction

Genome instability is a cell pathology in which chromosomes undergo alterations in the form of DNA breaks, mutations, rearrangements and loss at a high rate. In many cases, the mechanism responsible for genome instability implies a DNA replication failure. For this reason, genome instability and replication stress are two features tightly linked and are hallmarks of tumor cells [1, 2]. Chromosome duplication emerges thus like the most vulnerable process in the cell, so that events impairing progression of the replication fork (RF) have the potential of compromising genome integrity [3].

Apart from DNA damage generated by reactive oxygen species (ROS) and other natural genotoxic agents such as reactive aldehydes (RA), transcription is a major natural contributor to genome alterations. In the last decade evidence has accumulated that co-transcriptional R loops, structures formed by an RNA-DNA hybrid and a single strand DNA (ssDNA), may have an important role in the origin of genome instability [4–6]. From yeast to human cells, different factors play distinct roles in maintaining low levels of R loops along the genome. Importantly, mutations in such factors not only lead to accumulation of R loops above wild-type (WT) levels but also cause genome instability [7–13]. R loops, however, have been observed at different regions of the eukaryotic genome [14, 15] and have also regulatory roles in transcription [5].

Cells have two ways to limit R loops, those resolving them, such as RNase H or Senataxin, and those preventing their formation such as Topo I, the THO complex or the SRSF splicing factor, among other functions [4]. These functions serve to prevent genome instability by avoiding accumulation of R loops as a putative barrier to RF progression [16, 17], The observations that R loops trigger chromatin condensation and heterochromatin formation [5, 18, 19] suggest the possibility that chromatin compaction may be a major source of R-loop-mediated replication stress and genome instability [20], consistent with previous observations linking premature chromatin condensation and chromosome fragility [21]. Interestingly, factors like the yeast and human FACT chromatin reorganizing complex, which is crucial for RF progression through transcribed regions [22], and of the human BRCA1 and BRCA2 double-strand break repair (DSB) factors [23, 24] are also involved in R loop processing.

The fact that BRCA2/FANCD1 and BRCA1 directly or indirectly participate in the Fanconi Anemia (FA) pathway, involved in the repair of inter-strand crosslinks (ICLs) that block RF progression [25, 26] suggests that R loops may be an important contributor to genome instability in FA cells. To test this hypothesis we investigated the role of the FA pathway in resolving R loops and in protecting cells from R loop-mediated DNA breaks. Using human and murine cells defective in FANCD2 or FANCA and primary bone marrow cells derived from FANCD2 deficient mice, we validated our hypothesis. We propose that R loops accumulate in BRCA/FA- cells due to the incapacity of these cells to replicate R loop-containing regions.

## Results

### R loops accumulate in FANCA-/- and FANCD2-/- patient cell lines and knocked-down human cell lines

**Fig 1:**
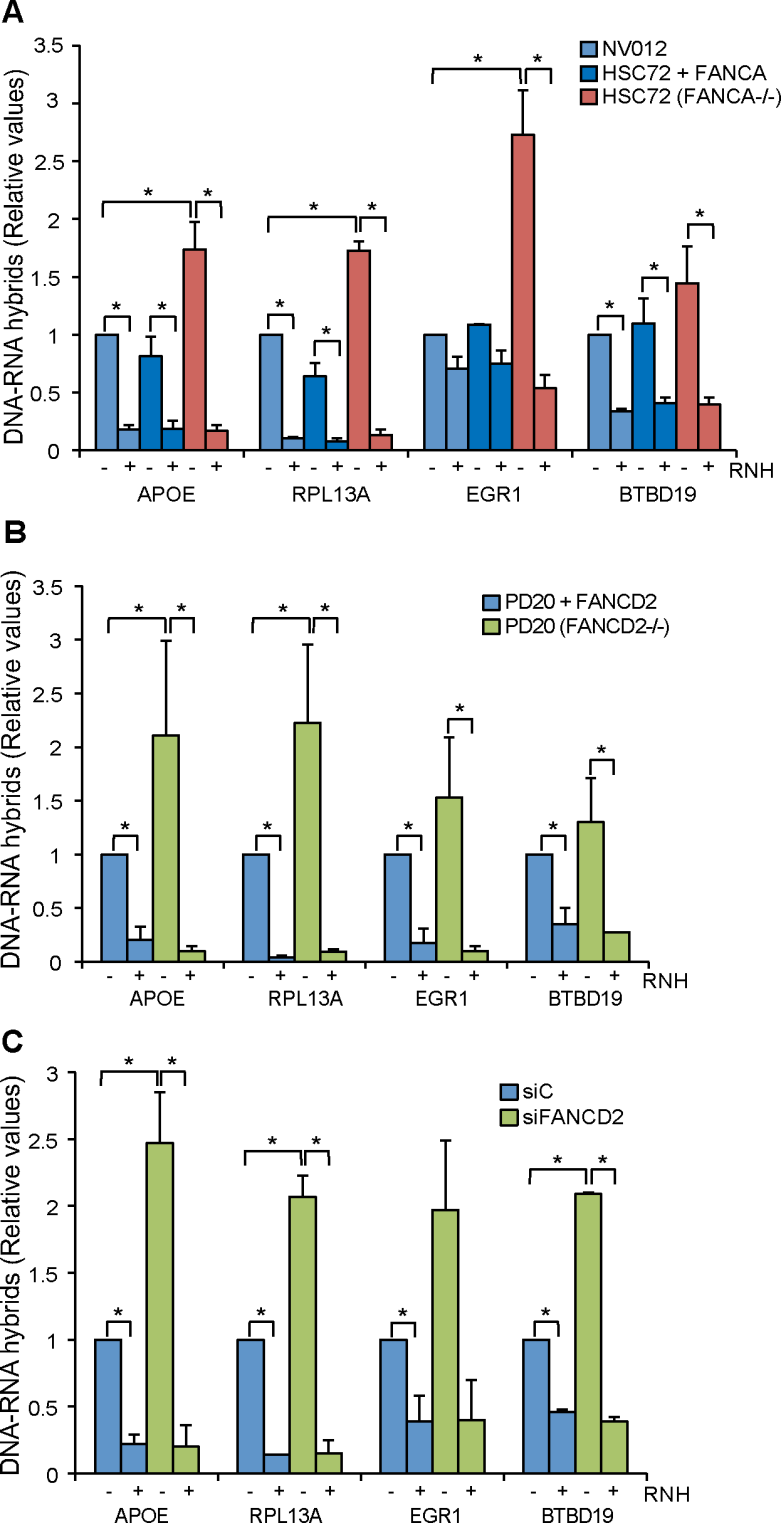
R loop accumulation in Fanconi Anemia-deficient patient cells. (**A**) DRIP-qPCR using the anti-RNA-DNA hybrids S9.6 monoclonal antibody, in FANCA-deficient human HSC72 lymphocytes and the corrected FANCA+ cells at *APOE*, *RPL13A*, *EGR1*, and *BTBD19* genes. Pre-immunoprecipitated samples were untreated (-) or treated (+) with RNase H (RNH) as indicated. Signal values of RNA-DNA hybrids immunoprecipitated in each region, normalized to input values and to the signal at the *SNRPN* negative control region are shown. Data represent mean ± SEM from three independent experiments. *, P < 0.05 (Mann-Whitney U test). (**B**) Relative amount of R loops in patient FANCD2-/- human PD20 cell line and the corrected PD20 FANCD2+/+ control at 4 different genes. Details as in (**A**). (**C**) Levels of DNA-RNA hybrids accumulated in actively transcribed genes in HeLa + siC, HeLa + siFANCD2 cells, as determined by DRIP-qPCR using the S9.6 monoclonal antibody with and without RNase H (RNH) treatment. Details as in (**A**).

To assay whether the FA pathway has a role in preventing or resolving R loops in human cells, we analyzed R loop accumulation in cells with dysfunctional FANCA or FANCD2 proteins or depleted of either of them (Fig 1). We performed DRIP-qPCR in four human genes, *APOE*, *RPL13A*, *EGR1* and *BTBD19* (S1 Fig) in well-established cell lines derived from Fanconi Anemia patients. We selected these four genes because they were identified as regions prone to form R loops and have been positively validated for the analysis of R loop accumulation [14, 22, 23].

We first determined the levels of R loops in wild-type lymphoblast cell line NV012 used as a reference control as well as in FANCA-/- lymphoblast patient cell line HSC72 and its corrected version [27]. Results clearly show that in all four genes tested, FANCA-/- cell lines accumulated R loops at a statistically significant higher level in *APOE*, *RPL13A* and *EGR1*; in *BTBD19* the R loops were also higher but to a lower level (Fig 1A). Importantly, when the samples were treated with RNase H that digests the RNA moiety of RNA-DNA hybrids, the levels of R loops dramatically decreased, confirming that indeed the signal detected was specific for RNA-DNA hybrids. The absolute amount of R-loop signal as a function of input DNA is also provided (S2 Fig). In all analyses, the *SNRPN* gene was used as negative control because it does not accumulate R-loops, as previously reported [22, 23], and to normalize the values of the four genes analyzed (Fig 1).

Next we assayed whether this result could be extended to another cell line from an FA patient. We used the PD20 human fibroblast cell line from a FANCD2-/- patient [28]. Again, there was a clear increase of R loop accumulation in all four genes analyzed, this accumulation being statistically significant in *APOE* and *RPL13A* (Fig 1B). Importantly, the R loop signal was dramatically and significantly reduced when samples were treated with RNase H. Therefore, we can conclude that cell lines of different tissues from patients with two different dysfunctional FA genes accumulate R loops.

**Fig 2:**
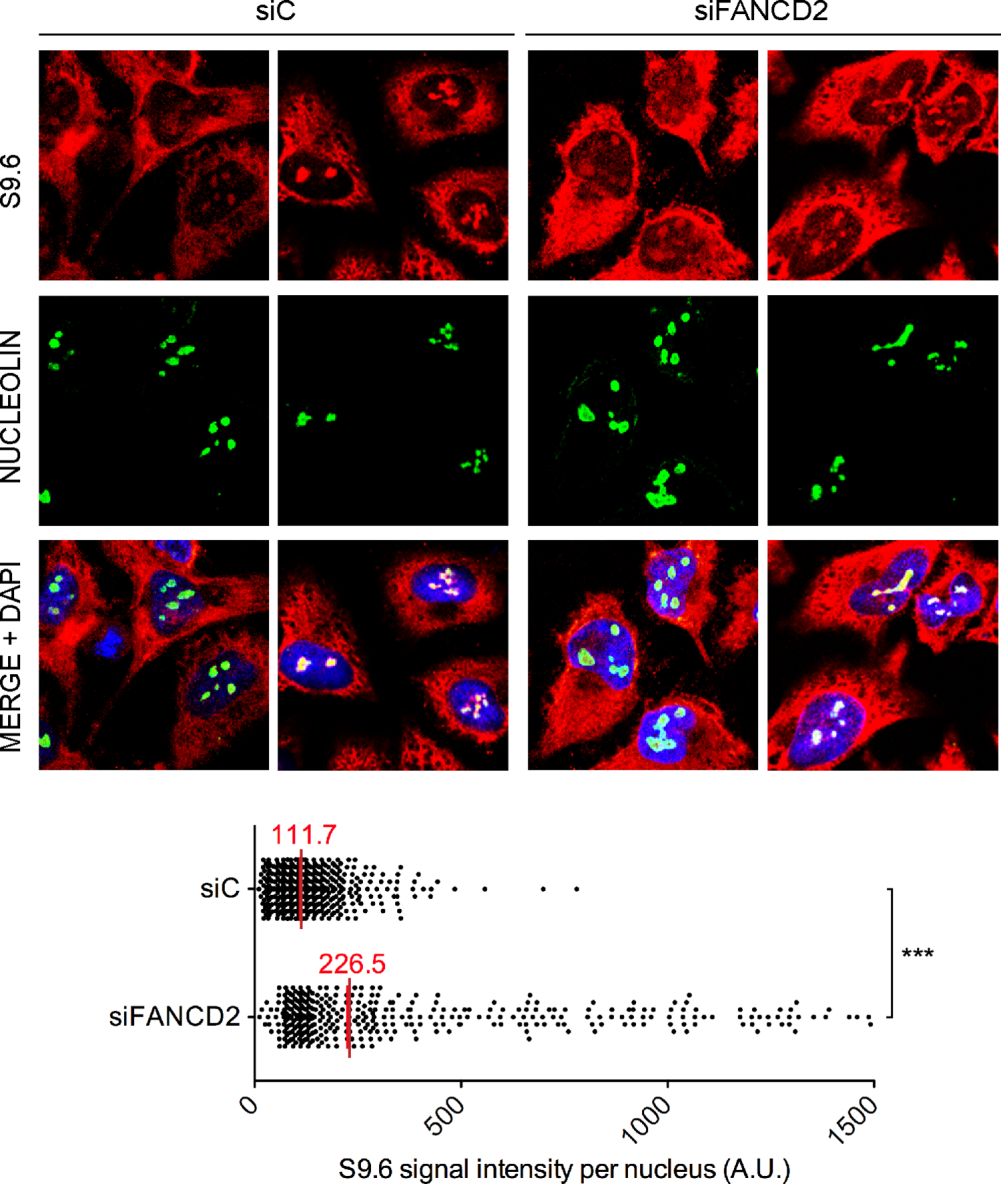
RNA-DNA hybrid accumulation in FANCD2-depleted HeLa cells. Immunostaining with S9.6 and nucleolin antibodies of siC and siFANCD2 HeLa cells. The graph shows the median of the S9.6 signal intensity per nucleus after nucleolar signal removal. More than 300 cells from two independent experiments were considered. ***, P < 0.001 (Mann-Whitney U test, two-tailed).

Finally, we tested whether this conclusion was also valid for HeLa cells depleted of FA proteins. We depleted cells of FANCD2 by siRNA (S3 Fig) and R loops accumulation was assayed in the same four human genes tested in patient cell lines. R loops clearly increased in siFANCD2 cell lines, up to 3 fold above the siC control levels (Fig 1C). The results confirm that a deficiency in the FA pathway, regardless of whether occurring in cells from human patients or in standard cell lines depleted of an FA factor by siRNA, leads to R loop accumulation. The similarity of results for the FANCD2-/- patient PD20 cell line and siFANCD2 depleted cells enabled us to use FANCD2-depleted HeLa cells as a reliable system to study the role of R loops in FA-deficient cells. In addition to demonstrate the presence of high levels of RNA-DNA hybrids as a consequence of *FANCD2* knockdown at the molecular level by DRIP, we also confirmed this fact at the cellular level by immunofluorescence (IF) (Fig 2).

### Murine FANCD2-deficient cells accumulate R loops

**Fig 3:**
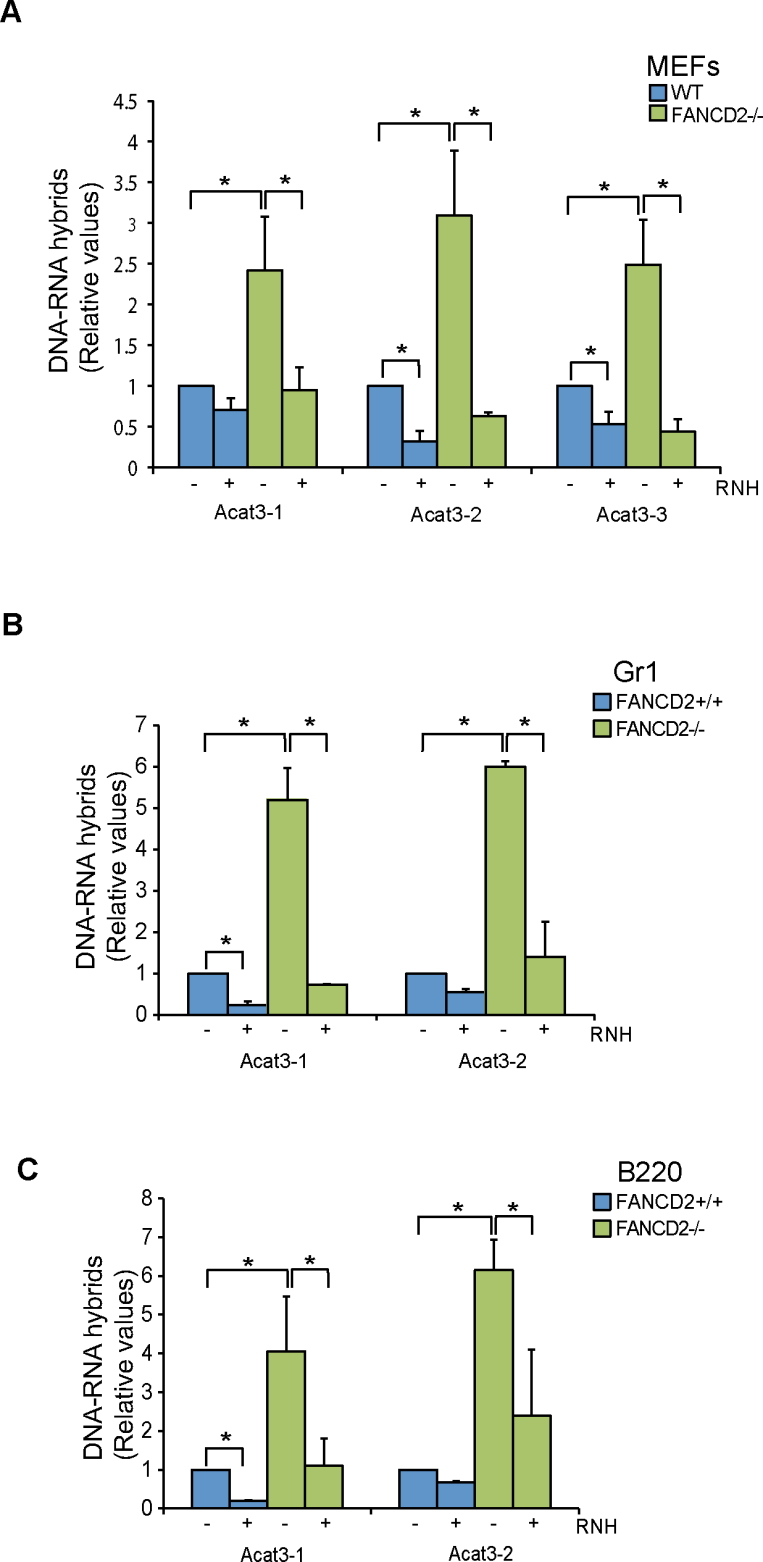
R loops in FANCD2-deficient murine cells. (**A**) Relative levels of RNA-DNA hybrids as determined by DRIP-qPCR in the *Acat3* gene at 3 independent regions in WT and FANCD2-/- MEFs with and without RNase H (RNH) treatment. (**B**) Relative levels of R loops as detected by DRIP-qPCR analysis at independent regions of the *Acat3* gene in WT and FANCD2-/- murine bone marrow Gr1+ cells with and without RNase H treatment. (**C**) Relative levels of R loops as detected by DRIP-qPCR analysis at independent regions of the *Acat3* gene in wild-type and FANCD2-/- murine bone marrow B220 cells with and without RNase H (RNH) treatment. Other details as in Fig 1.

So far we have demonstrated that R loops accumulate in transformed human cells. Next we assayed R loop accumulation in murine embryonic fibroblasts (MEFs) obtained from mice defective in FANCD2. We performed DRIP-qPCR analyses in three different regions of the *Acat3* gene (Acat3-1, Acat3-2 and Acat3-3) formerly annotated as *AIRN locus* (S4 Fig), which have been shown to be reliable for R loop detection in murine cells, as assayed by non-denaturing bisulfite treatment combined to RNase H digestion [15]. DRIP-qPCR in FANCD2-/- MEFs reveals a statistically significant increase of up to 3 fold in R loop accumulation compared to wild-type MEFs (Fig 3A). As expected, the signals decreased when MEFs were treated with RNase H, confirming that the signal detected was specific for R loops.

Next we addressed whether R loops physiologically accumulated in bone marrow cells from FANCD2-/- mice. We analyzed R loop accumulation in the Acat3-1 and Acat3-2 regions of myeloid Gr1+ and lymphoid B220+ committed cells from FA mice by DRIP-qPCR and observed that again R loops were clearly accumulated, at least 5 fold over the levels observed in WT mice (Fig 3B and 3C). As expected, the detected signal was clearly decreased by RNase H treatment.

Our results both at molecular and cellular levels indicate, therefore, that human cells deficient in the FA pathway accumulate R loops, regardless of the cell type analyzed, and the same occurs in bone marrow cells from FANCD2-deficient mice.

### DNA breaks accumulated in FA cells are mediated by R loops

**Fig 4:**
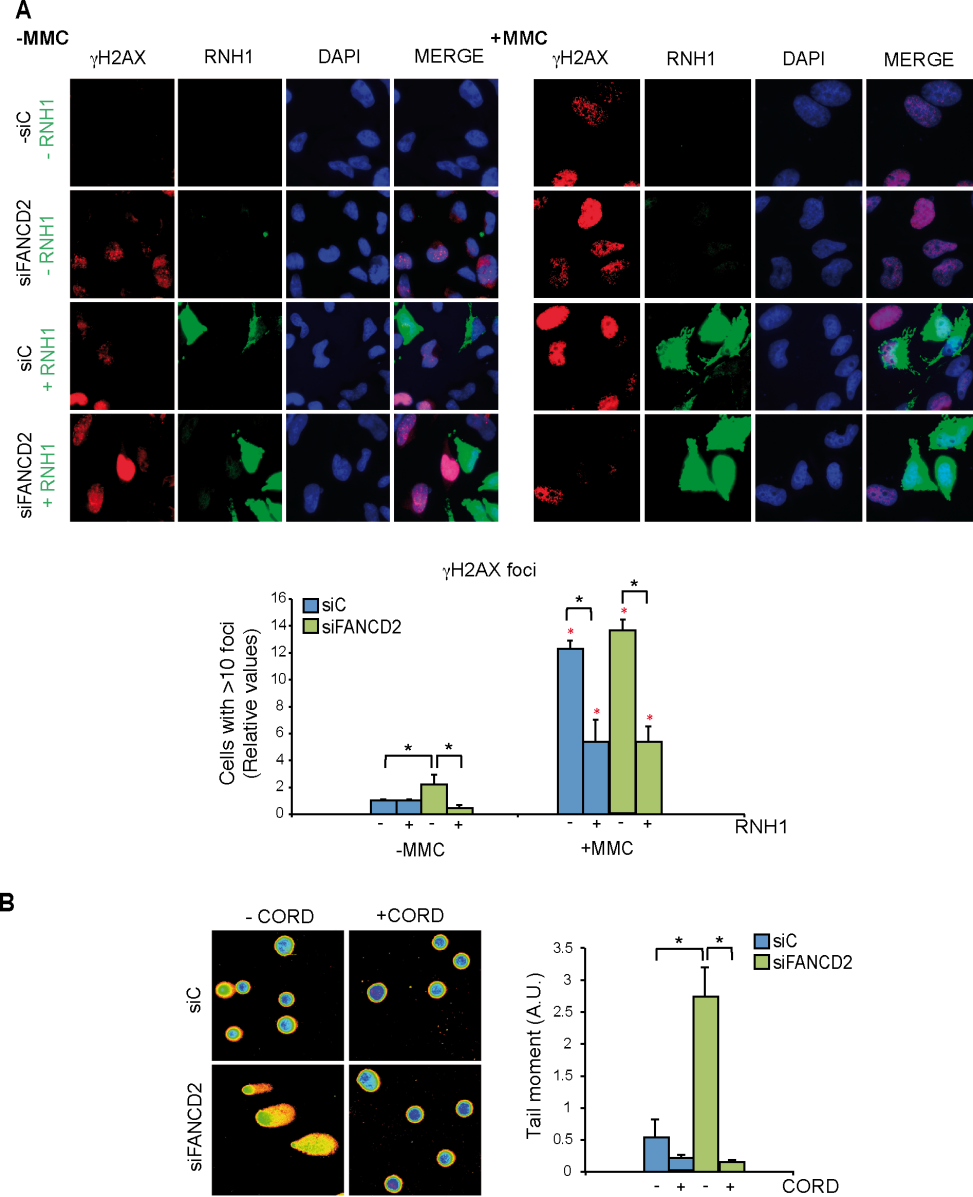
Genome instability in FANCD2-depleted human cells. (**A**) Detection of γH2AX foci by IF in siC and siFANCD2 HeLa cells transfected with pcDNA3 (-RNH1) or pcDNA3-RNaseH1 (+RNH1) for RNase H1 overexpression and either untreated or treated for 16 h with 80 ng/ml mitomycin C (MMC). Nuclei were stained with DAPI. The graph shows the quantification of the relative amount of cells containing >10 γH2AX foci with respect to the siC in each case. More than 100 cells overexpressing RNase H1 (positive-stained) or more than 100 cells of mixed population transfected with the empty vector were counted in each of the three experiments. Data represent mean ± SEM from three independent experiments. The red asterisks refer to the comparison of each MMC-treated samples versus its own untreated sample. *, P < 0.05 (Mann-Whitney U test). As a reference the percentage of cells with ≥10 γH2AX foci is >75% in siC cells treated with MMC (**B**) DNA breaks measured by single-cell gel electrophoresis (comet assay) of siC and siFANCD2 HeLa cells treated or untreated for 4 h with 50 μM cordycepin. The graph shows the median comet tail moment. More than 100 cells were counted in each of the three experiments. Data represent mean ± SEM from three independent experiments. *, P < 0.05 (Student’s t-test).

Once demonstrated that both human and murine cells defective in FA genes accumulate R loops, we investigated the functional impact of R loops in cells with a defective FA pathway. For this we used the FANCD2-depleted HeLa cells. First we wondered whether the accumulation of double strand breaks (DSBs) in siFANCD2 cells was related to R loop accumulation. We assayed DSBs indirectly by detection of γH2AX foci by immunofluorescence in cells transfected with a control or an RNase H1 overexpressing plasmid. Upon knockdown of FANCD2 a significant increase in γH2AX foci formation was observed by quantifying the number of cells with more than 10 foci (Fig 4A, left panel). Importantly, those high levels of γH2AX foci were strongly reduced by RNase H1 overexpression, confirming that γH2AX formation in FANCD2-depleted cells is R loop-dependent. Since the overexpressed RNase H1 protein localized both in cytoplasm (mitochondria) and nucleus, to prove that the observed effect was specific of the nuclear function of RNase H1 we performed the same experiment using the truncated version of the RNase H1 that lacks the mitochondrial localization signal and localizes only into the nucleus (S5 Fig) [29]. As expected, overexpression of the nuclear form of RNase H1 reduced the γH2AX foci accumulation caused by FANCD2 depletion.

As the hallmark phenotype of FA-deficient cells is sensitivity to inter-strand crosslinking (ICL) agents such as mitomycin C (MMC) we reasoned that MMC might not only generate DNA-DNA ICLs but also RNA-DNA ICLs that would eventually lead to DNA breaks. Therefore, we determined whether MMC-induced γH2AX foci in siFANCD2 HeLa cells were also mediated by R loops. MMC clearly increased γH2AX foci both in siC and siFANCD2 cells as seen by IF and quantification of cells with more than 10 foci, whose value increased 12 to 14 fold above those of the untreated cells (Fig 4A, right panel) reaching >75% in siC MMC-treated cells and >85% siFANCD2 MMC-treated cells. Importantly, however, a large fraction of these MMC-induced foci were reduced in cells transfected with the RNase H1-overexpressing plasmid, confirming that they were R loop-dependent. This conclusion is further supported by the observation that MMC-induced γH2AX foci were also significantly reduced by RNase H1 overexpression in MEFs FANCD2-/- (S6 Fig). Consistent with the partial R-loop dependency of γH2AX foci, DNA-RNA hybrids accumulate at higher levels in cells treated with MMC, as detected by a significant increase in S9.6 signal both in siC and siFANCD2 depleted cells (S7 Fig).

The statistically significant reduction of MMC-induced breaks in both siC and siFANCD2 cells by RNase H1 overexpression suggests that MMC may induce ICLs also at RNA-DNA hybrids as a cause of its DNA damage capacity, and that such RNA-DNA hybrids may be a relevant source of MMC-induced DNA breaks in siFANCD2 cells. Although we cannot formally discard the possibility that ICLs did not form at RNA-DNA hybrids, but instead hybrids could divert limiting-DNA repair factors from ICLs, so that RNase H1 would promote ICL repair indirectly by degrading R-loops and releasing such repair factors, there is no chemical basis to assume that ICLs cannot form between RNA and DNA strands.

If siFANCD2 cells accumulate DNA breaks dependent on co-transcriptional R loops, the breaks should be transcription-dependent. To assay this, we performed single cell electrophoresis or comet assay in cells incubated with cordycepin, a specific inhibitor of adenine incorporation into the nascent RNA. DNA breaks were clearly reduced in the presence of cordycepin in both siC and siFANCD2 cells (Fig 4B *)*, suggesting that a large portion of DNA breaks in cells are mediated by transcription, as expected (reviewed in [30]). Importantly, a significant 5-fold increase in DNA breaks was observed in siFANCD2 cells compared to control cells, that was completely suppressed by cordycepin (Fig 4B *)*. Therefore, DNA breaks accumulated in siFANCD2 cells are transcription-dependent, consistent with them being mediated by co-transcriptional R loops.

### FANCD2 foci are R loop-dependent

**Fig 5:**
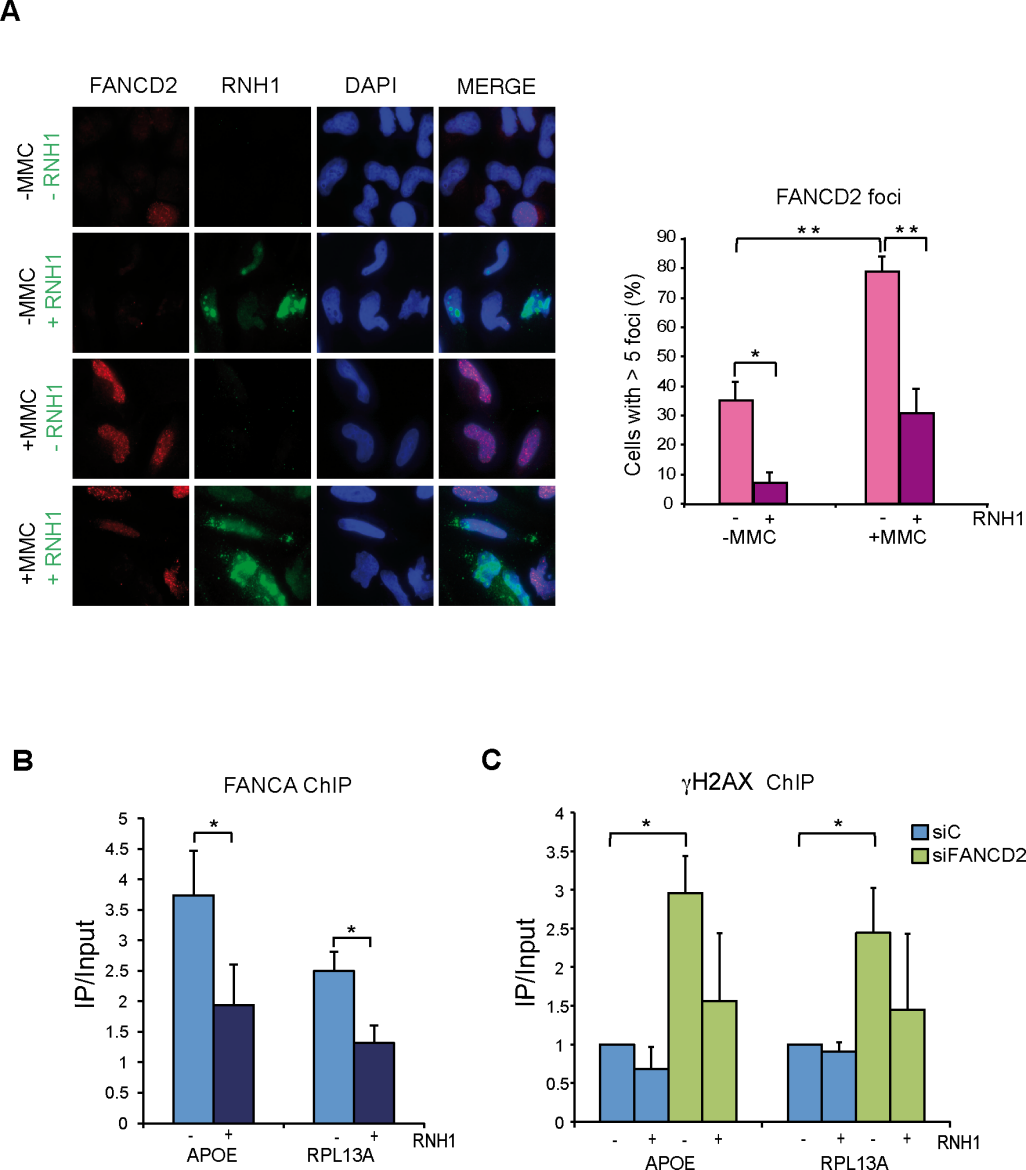
FANCD2 foci assemble in a R-loop dependent manner. (**A**) Immunofluorescence of FANCD2 in HeLa cells with or without RNase H1 overexpression treated or untreated for 16 h with 40 ng/ml MMC and then released for 9 h. More than 100 cells overexpressing RNase H1 (positive-stained) or more than 100 cells of mixed population transfected with the empty vector were counted in each of the three experiments. The graph shows the quantification of the percentage of cells. containing >5 FANCD2 foci. Data represent mean ± SEM from three independent experiments. *, P < 0.05 (Student’s t-test). **, P < 0.01 (Student’s t-test). Other details as in Fig 4. (**B**) ChIP analysis of FANCA recruitment in HeLa cells with or without RNase H1 overexpression. Signal values of DNA immunoprecipitated in each region, normalized to input values and to the signal without antibody are shown. Data represent mean ± SEM from four independent experiments. *, P < 0.05 (Mann-Whitney U test). (**C**) ChIP analysis of γH2AX in siC and siFANCD2 HeLa cells with or without RNase H1 overexpression. Data represent mean ± SEM from four independent experiments. *, P < 0.05 (Mann-Whitney U test).

As the FA pathway repairs ICLs that impede normal RF progression [31] we reasoned that if ICLs are formed between the RNA and DNA strands, the FA core complex should accumulate at sites containing RNA-DNA hybrids. Therefore, we expected that removal of R loops by RNase H1 overexpression reduced the accumulation of FA foci at the sites of putative RF blockages. To test this possibility we performed IF with anti-FANCD2 antibody in HeLa cells transfected with the plasmid overexpressing RNase H1 as well as the empty plasmid in cells untreated and treated with MMC. As can be seen in Fig 5A, a significant increase of FANCD2 foci was observed after MMC treatment. Importantly, overexpression of RNase H1 drastically reduced FANCD2 foci both in MMC-treated and untreated cells. To exclude the possibility of an indirect effect of RNase H1 overexpression that could slow down proliferation and indirectly the activation of the FA pathway, we determined the effect of RNase H1 overexpression on cell cycle progression by measuring via BrdU incorporation and FACS analysis the percentage of cells in S phase (S8 Fig). We found that 24 hours after plasmid transfection there was no difference in the amount of cells in S phase in RNase H1 overexpressing cells with respect to control cells, which rules out a major impact of RNase H1 on cell proliferation to explain our results.

This result is consistent with the FA core complex locating at RFs blocked at R loop sites and supports that MMC causes RNA-DNA ICLs, indicating that the FA pathway plays a key function assisting the repair of RFs blocked at R loop-containing sites. To prove that the FA pathway acts at the sites where RNA-DNA hybrids are accumulated we performed ChIP of the FA core complex protein FANCA. Using anti-FANCA antibody we found that the FA core complex is indeed recruited to the genes that we had shown to accumulate RNA-DNA hybrids in FA deficient cells (Fig 5B). Finally, to demonstrate the functional link between the site of action of these FA complexes and DNA damage, we assayed whether γH2AX was enriched at genes that accumulate RNA-DNA hybrids in the absence of a functional FA pathway in an R loop-dependent manner. ChIP analyses with anti-γH2AX antibody confirmed that this was the case (Fig 5C). RNase H1-sensitive γH2AX signals were significantly higher in FANCD2-/- cells, confirming a physical link of R-loops with the regions at which DNA damage and the FA proteins are found.

## Discussion

We demonstrated using different cell lines defective in FANCD2 or FANCA from either human patients or HeLa and primary bone marrow murine cells, that FA cells accumulate R loops. Using siFANCD2 HeLa cells, we demonstrated that the increase in DNA breaks is strongly reduced by RNase H1 overexpression and transcription inhibition. The results indicate that the FA pathway plays an important role in protecting cells from naturally formed R loops and that R loops are a major source of DNA breaks in FA cells. This not only occurs in untreated cells, but also in cells treated with the ICL agent MMC (Figs 4 and 5). RNA-DNA hybrids seem to be a major source of RF blockage that requires the FA pathway for replication resumption and repair. Proper replication in FA+ cells would contribute to prevent R loop accumulation. Consistently, a high increase in R loops was observed in highly proliferative bone marrow tissues and MEFs from replication-impaired FANCD2-/- mice (Fig 3).

We have recently shown that BRCA2- and BRCA1- cells accumulate R loops and that an important fraction of the DNA breaks generated in these cells could be suppressed by RNase H1 overexpression [23]. A role of BRCA1 in R loop resolution is supported by its ability to recruit the RNA-DNA helicase SETX to DNA [24, 32]. As BRCA2 binds ssDNA [33] and protects RFs, avoiding their collapse [34, 35], a major role for BRCA2 in preventing R loop accumulation could be mediated by replication, without excluding additional putative roles of BRCA2 and BRCA1 in non-replicating cells. The FA pathway is involved in the repair of ICLs [36], and the fact that BRCA2/FANCD1 is a member of the FA pathway and BRCA1 has an FA-associated function opened the possibility that the FA pathway played a relevant role in removing R loops via the replication of R-loop-containing regions [23]. FANCA belongs to the FA core complex, FANCD2 being the main switcher that activates the pathway after monoubiquitination. Our work with FANCD2-/- and FANCA-/- cells therefore demonstrates the need of the FA pathway to remove R loops or R loop-associated DNA damage, including presumably RNA-DNA ICLs (Figs 1–4). This work also suggests that R loop accumulation might be a potential driver of bone marrow failure and haematopoietic stem cell attrition seen in FA mice deficient in aldehyde catabolism, but further work is needed to verify this hypothesis [37].

**Fig 6:**
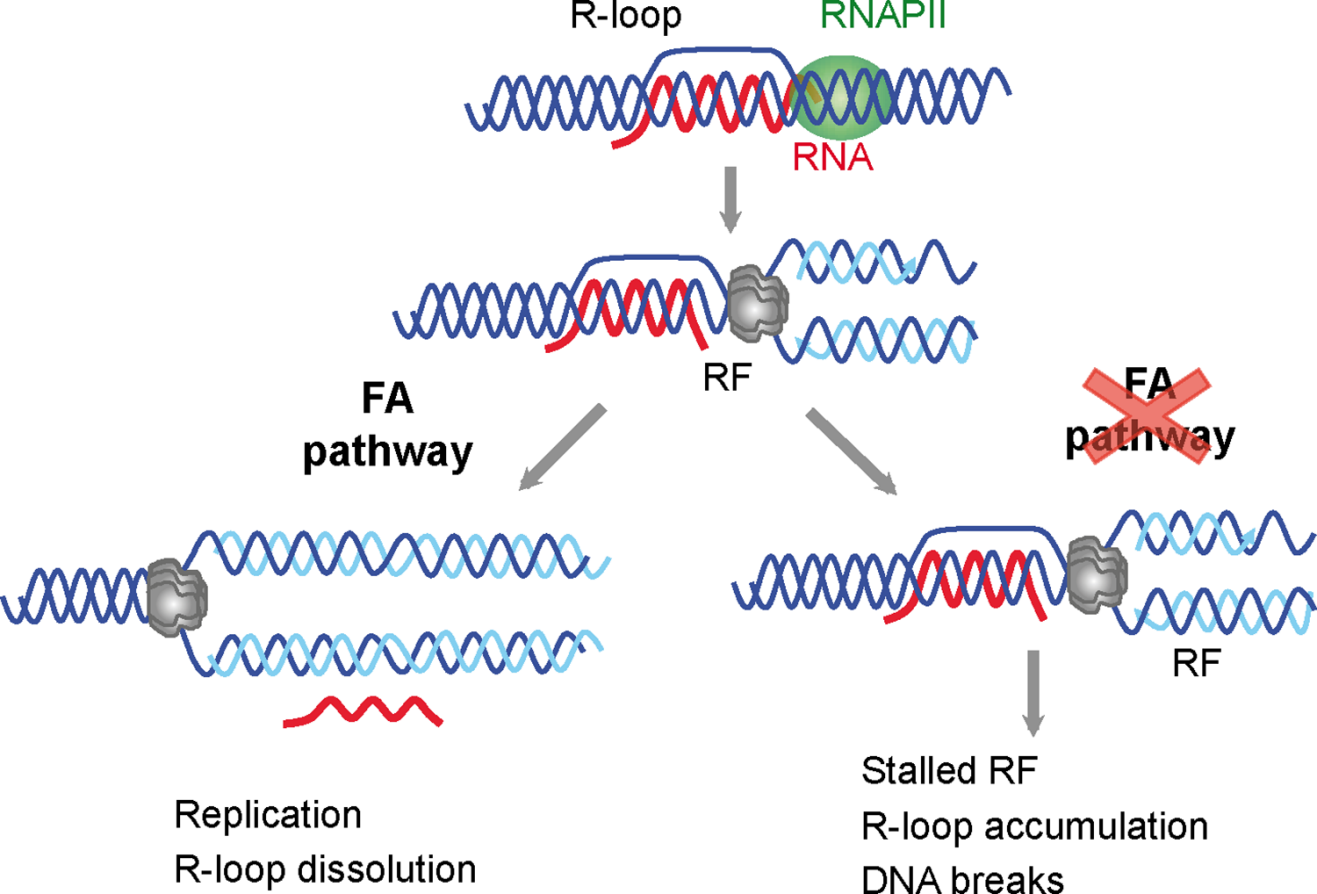
Model for a role of the FA pathway in preventing R-loop accumulation. The model explains the role of the FA pathway in preventing R loop-mediated genome instability. The FA pathway prevents R loop accumulation that hampers replication fork (RF) progression and leads to DNA breaks. For clarity, only the MCM helicase is depicted at the replication fork.

Our study suggests that in addition to ribonuclease H and RNA-DNA helicases, R loops might also be resolved during replication/repair. The observation that the FACT chromatin reorganizing complex is involved in RF progression preferentially when transcription is active and that FACT dysfunction leads to R loop accumulation in yeast and human cells, indicate indeed that R loops are a main source of genome instability in cells unable to properly replicate through R loop-containing regions [22]. The role of the FA pathway would be critical for progression of RFs stalled at either R loops or RNA-DNA ICLs. By repairing the R loop-dependent RF block, the R loop would be removed. This is consistent with the observations that FANCD2 foci formed in MMC-treated and untreated cells are strongly reduced by RNase H1 overexpression (Fig 5A), and that FANCA is recruited to R loop-forming genes in an RNA-DNA hybrid-dependent manner (Fig 5B). In addition, DNA damage accumulation in FANCD2-depleted cells specifically takes place at R-loop forming genes (Fig 5C), strengthening the hypothesis that the FA core complex assembles at sites where R loops block the progression of RFs and prevents R loop-dependent DNA damage, as proposed in our model (Fig 6).

R loops may thus constitute a major source of replication stress and genome instability. These are features commonly found in cancer cells and cells lacking a functional FA pathway that will not be able to resume replication through R-loop containing regions. This study, therefore, not only provides evidence that co-transcriptional R loops are major sources of replication stress, but also demonstrates that the FA pathway plays a crucial role in the repair of R loop-mediated damage or RF blockage (Fig 6). We propose that the action of FA during replication allows the removal of the R loop, whereas in FA cells, the block persist and therefore R loops are accumulated and DNA breaks arise. Knowing of the ability of R loops to trigger chromatin condensation [18] it would be certainly interesting to assay in the future the contribution of chromatin condensation to this phenomenon.

## Materials and Methods

### Human cell culture and transfection

HeLa cells were cultured in Dulbecco’s modified Eagle’s medium (DMEM; GIBCO, Thermo Scientific, Waltham, MA) supplemented with 10% heat-inactivated fetal bovine serum at 37°C (5% CO_2_). Transient transfection of siRNA was performed using DharmaFECT1 (Dharmacon) according to the manufacturer’s instructions. Lipofectamine 2000 (Invitrogen, Carlsbad, CA) was used for plasmid transfection. All assays were performed 48 h after siRNA transfection plus 24 h after plasmid transfection as described previously [38]. The plasmids used were the following: pcDNA3, an empty vector; pcDNA3-RNaseH1, containing the full length RNase H1 cloned into pcDNA3 [39]; pEGFP, a vector expressing GFP, and pEGFP-M27, containing the GFP-fused RNase H1 lacking the first 26 amino acids responsible for its mitochondrial localization cloned into pEGFP [29].

Human NV012 (WT), HSC72 (FA-A) and HSC72+FANCA (FANCA-corrected) EBV-immortalised lymphoblastoid cell lines [27] were cultured in RPMI 1640 (GIBCO) supplemented with 15% heat-inactivated fetal calf serum under standard culturing conditions. Human transformed fibroblasts PD20 (FANCD2^-/-^) and PD20 corrected (PD20 retrovirally corrected with pMMp-FANCD2 cDNA) [28] were grown in DMEM (GIBCO) supplemented with 15% heat-inactivated fetal calf serum as previously described [40, 41].

Mitomycin C (MMC, M4287, Sigma-Aldrich) was used to a final concentration of 80 ng/ml for 16 h for detection of γH2AX foci, 250 ng/ml for 5 h for S9.6 inmunofluorescence and 40 ng/ml for 16 h and then released for 9 h for FANCD2 foci.

### Mouse maintenance and murine cell cultures

*Fancd2* ^*-/-*^ mice (*Fancd2* ^tm1Hou^, MGI code: 2673422, backcrossed into C57BL/6 background for at least 11 generations), obtained from K.J. Patel [42, 43], were maintained in a conventional mouse facility. All animal experiments undertaken in this study were performed under the approval of the EU Directive 2010/63EU, Spanish law RD53/2013 and the Hospital Virgen del Rocio Ethical Review Committee. Timed matings between *Fancd2* ^*+/−*^ males and females were set up. Females were checked for the presence of a vaginal plug the following morning, and considered to be at day E0.5 of pregnancy. Pregnant females were sacrificed at E13.5, uteruses removed and embryos dissected. Murine embryonic fibroblasts (MEFs) cultures were obtained, genotyped and transformed using a lentiviral vector pLOX-Ttag-iresTK (addgene 12246). Clones were isolated and expanded.

Murine bone marrow cells from femora and tibias were obtained by flushing in 2 mls of PBS+3% fetal bovine serum. Cells were enumerated using trypan blue 0.2% in a TC20 Automated Cell Counter (Bio-Rad). Biotinylated B220 (cloneRA3-6B2), Gr1 (Clone RB6-8C5) were obtained from BDBioscience. Cells were enriched using Streptavidin-bound magnetic particles (BD IMag) according to manufacturer instructions.

### Immunofluorescence and single-cell electrophoresis

For the analysis of DNA damage foci, immunofluorescence was performed as described previously [38]. FANCD2 IF was performed as described previously [44] with minor modifications. In brief, cells were pre-permeabilized with 0.25% Triton X-100 in PBS for 1 minute on ice and then fixed with 2% formaldehyde in PBS. After blocking with 3% BSA in PBS, cells were incubated with the anti-FANCD2 (1:100 dilution) and the anti-RNASEH1 (1:400 dilution) followed by the secondary antibody conjugated with Alexa 488 and Alexa 546. DNA was stained with DAPI. In pre-permeabilized cells the overexpressed RNase H1 stained only nucleus and nucleoli because the rest of the protein had been washed out. S9.6 (hybridoma cell line HB-8730) immunofluorescence was performed as previously described [45] using secondary antibodies conjugated with Alexa 488 and Alexa 647. Images of IF and single-cell electrophoresis were acquired with a Leica DM6000 microscope equipped with a DFC390 camera (Leica). Images of S9.6 immunofluorescence were acquired with a Leica TCS SP5 confocal microscope. Data acquisition was performed with LAS AF (Leica). Images were captured at ×63 (IF) and ×10 (comet assay) magnification. Metamorph v7.5.1.0 software (Molecular Probes) image analysis software was used to quantify foci and nuclear S9.6 signal intensity.

Comet assay was performed as described [22] using a commercial kit (Trevigen, Gaithersburg, MD, USA) following the manufacturer’s protocol. Means and SEM (Standard Error of the Mean) from three independent experiments were obtained and are shown in each case. Comet tail moments were analyzed using Comet-score software (version 1.5).

Anti-γH2AX (clone JBW301; Upstate), Anti-RNASEH1 (15606-1-AP; Proteintech), Anti-FANCD2 (sc-20022; Santa Cruz, Dallas, TX), anti-β-Actin (ab8226, Abcam, Cambridge, UK), anti-Vinculin (V9264; Sigma-Aldrich) and anti-nucleolin (ab50279 Abcam) antibodies were used.

### DNA-RNA immunoprecipitation (DRIP)

DRIP assays were performed as described [22], with the exception of the DRIP conducted in MEFs, in which double amount of RNase H was used. RNA-DNA hybrids were immunoprecipitated using the S9.6 antibody from gently extracted and enzymatically digested DNA, treated or not with RNase H [15]. Quantitative PCR was performed at the indicated regions (S1 and S4 Figs). The relative abundance of RNA-DNA hybrid immunoprecipitated in each region was normalized to the signal at the negative control region *SNRPN* gene in human cell lines. All experiments were performed in triplicate; average and SEM of results are provided.

### Chromatin immunoprecipitation (ChIP)

HeLa cells were transfected with the corresponding siRNAs and 48h after siRNA transfection, they were transfected with either the RNase H1-coding plasmid pEGFP-M27 or the control plasmid pEGFP-C1. After 72 h of siRNA transfection, cells were crosslinked and processed for ChIP using standard procedures with minor modifications as previously described [23]. Anti-FANCA (Bethyl Laboratories) or anti-γH2AX (clone JBW301; Upstate) previously conjugated with Dynabeads Protein A (Life Technologies) were used to immunoprecipitate chromatin.

### FACS analysis

Cells were pulse-labeled with BrdU 10 μM added directly to the growing medium for 20 min, harvested, fixed with 70% ethanol in PBS and incubated on ice for 1 h. Cells were then treated with 2 N HCl 0.5% Triton X-100 for 30 min at room temperature, then with 0.1 M Sodium tetraborate pH 8.5, washed once with washing buffer (1% BSA 0.1% Triton X-100 in PBS), and incubated for 1 h in the same buffer containing 1:25 anti-BrdU antibody conjugated with Alexa Fluor 488 (B35139, Invitrogen) and 0.5 μg/μl RNase A. After one wash with washing buffer, cells were resuspended in PBS containing 100 ng/ml propidium iodide to counterstain DNA for 30 min and examined by flow cytometry (FACSCalibur; BD).

## Supporting Information

S1 Fig (A) Positions along the genes of the amplicons used. (B) Sequences of the primers used to perform qPCR. (TIF)

S2 Fig Signal values of RNA-DNA hybrids immunoprecipitated in each region normalized to input values in (A) FANCA-/- human HSC72 lymphocytes and the corrected FANCA+/+ cells; (B) in FANCD2-/- human PD20 cell line and the corrected PD20 FANCD2+/+ control and (C) in siFANCD2 transfected HeLa cells and siC transfected control cells. A.U., Arbitrary Units.
(TIF)

S3 Fig Western blot showing FANCD2 expression in siRNA transfected HeLa cells. The amount of β-Actin protein was used as a loading control
(TIF)

S4 Fig (A) Positions along the gene of the amplicons used. (B) Sequences of the primers used to perform qPCR. (TIF)

S5 Fig Detection of DNA breaks by IF of γH2AX in siC and siFANCD2 HeLa cells transfected with pEGFP (-RNH1) or pEGFP-M27 (+RNH1) for GFP-RNase H1 overexpression and its nuclear localization. The graph shows the quantification of the relative amount of cells containing >5 foci with respect to the siC in each case. Data represent mean ± SEM from three independent experiments. *, P < 0.05 (Mann-Whitney U test).
(TIF)

S6 Fig γH2AX foci in FANCD2-/- MEFs treated (+MMC) or untreated (-MMC) with 80 ng/ml mitomycin C (MMC) for 16 h and transfected with pcDNA3 (-RNH1) or pcDNA3-RNase H1 (+RNH1). The graph shows the quantification of the relative amount of cells containing >10 foci with respect to the untreated (-MMC) cells. Data represent mean ± SEM from three independent experiments. * P < 0.05 (Mann-Whitney U test).
(TIF)

S7 Fig RNA-DNA hybrid accumulation detected by immunostaining with S9.6 and nucleolin antibodies in siC and FANCD2-depleted HeLa cells untreated or treated for 5 h with 250 ng/ml mitomycin C (MMC). The graph shows the median of the S9.6 signal intensity per nucleus after subtraction of the nucleolar signal. More than 300 cells from four independent experiments were considered. ***, P < 0.001 (Mann-Whitney U test, two-tailed).
(TIF)

S8 Fig FACS profile of HeLa cells transfected with the RNase H1-coding plasmid (pRNH1) or with the empty plasmid (pcDNA3). The graph shows the quantification of the percentage of cells in S phase. Data represent mean ± SEM from three independent experiments. Western blot shows the overexpression of RNase H1. The amount of Vinculin protein was used as a loading control.
(TIF)
