# Supplementary figures and images for: The Fanconi Anemia Pathway Protects Genome Integrity from R-loops
Source: PLoS Genet. 2015 Nov 19;11(11):e1005674. doi: 10.1371/journal.pgen.1005674 (PMC4652862; doi:10.1371/journal.pgen.1005674)

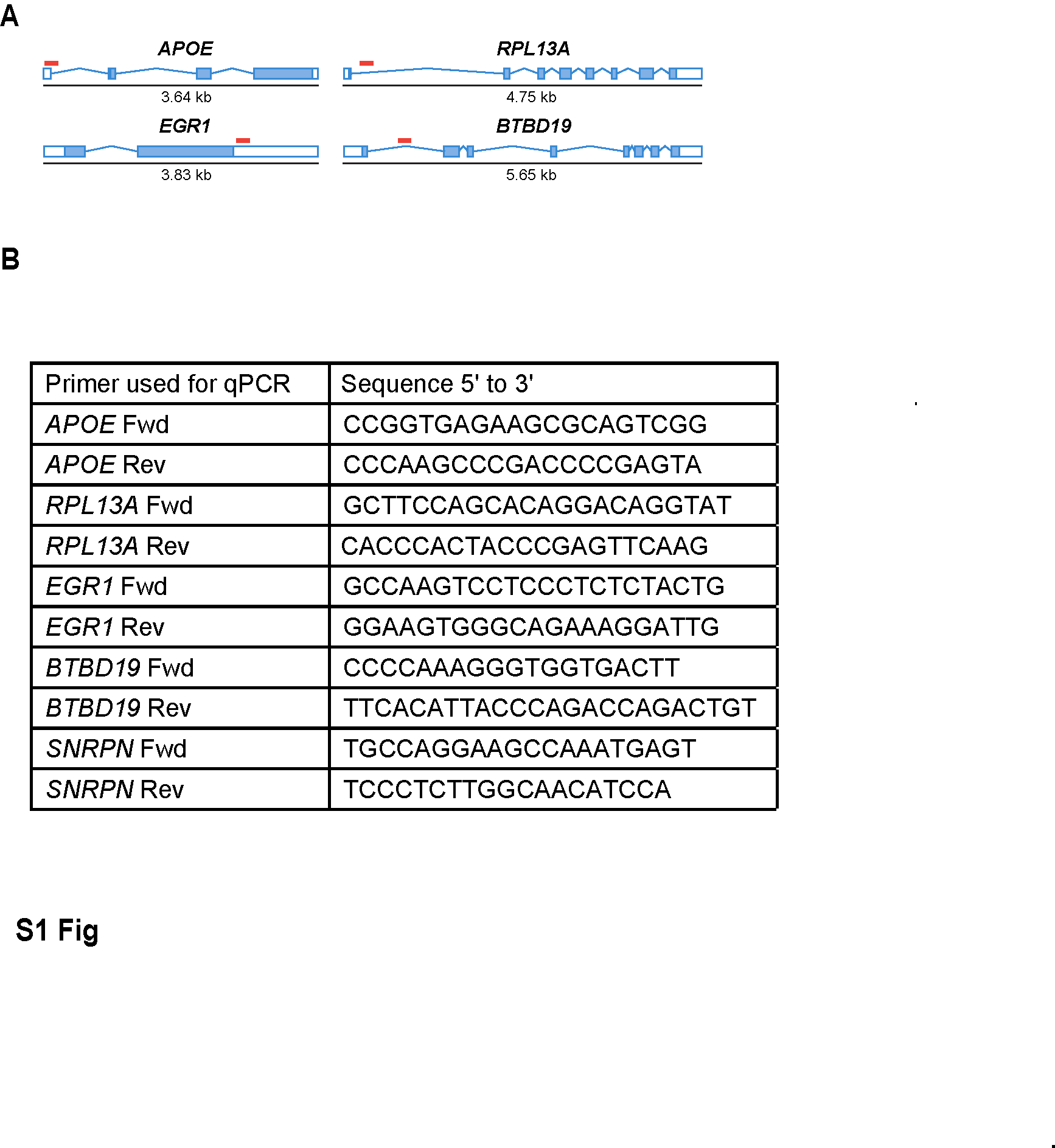

Supplement: S1 Fig — (TIF) [file pgen.1005674.s001.tif]

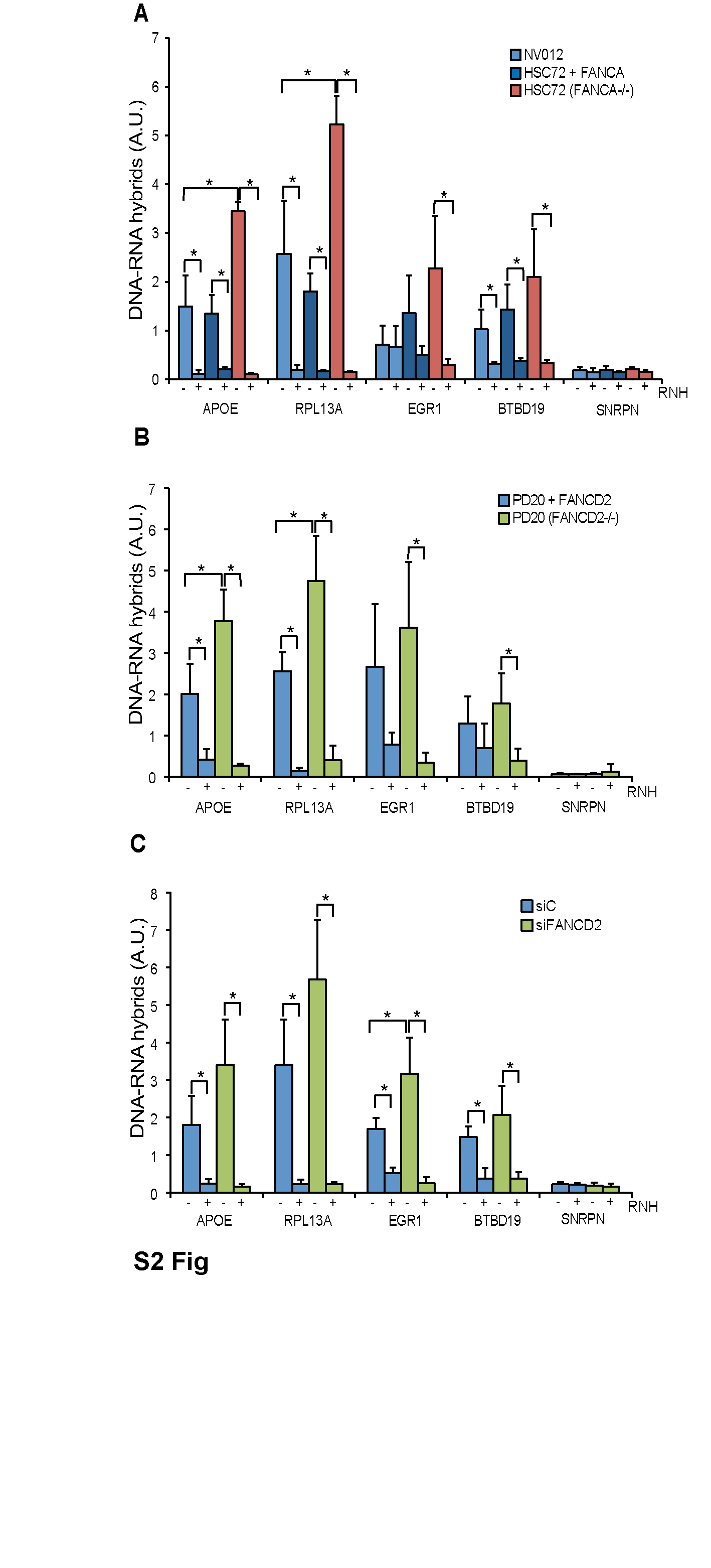

Supplement: S2 Fig — A.U., Arbitrary Units. (TIF) [file pgen.1005674.s002.tif]

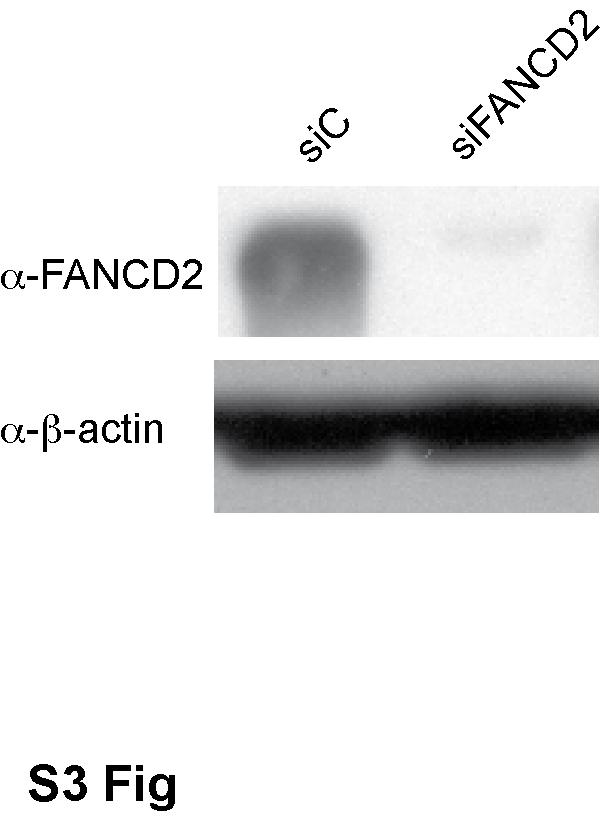

Supplement: S3 Fig — The amount of β-Actin protein was used as a loading control (TIF) [file pgen.1005674.s003.tif]

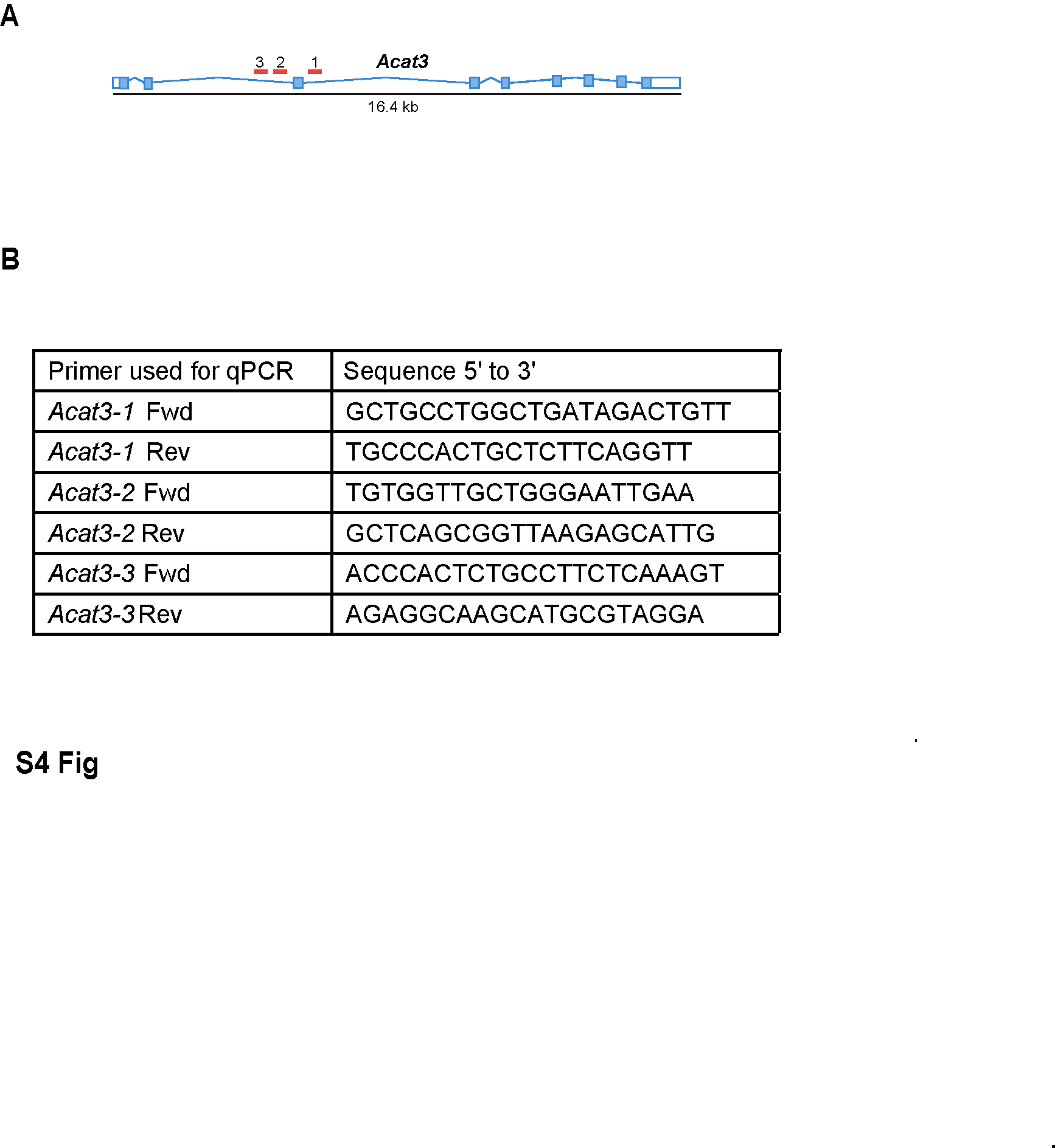

Supplement: S4 Fig — (TIF) [file pgen.1005674.s004.tif]

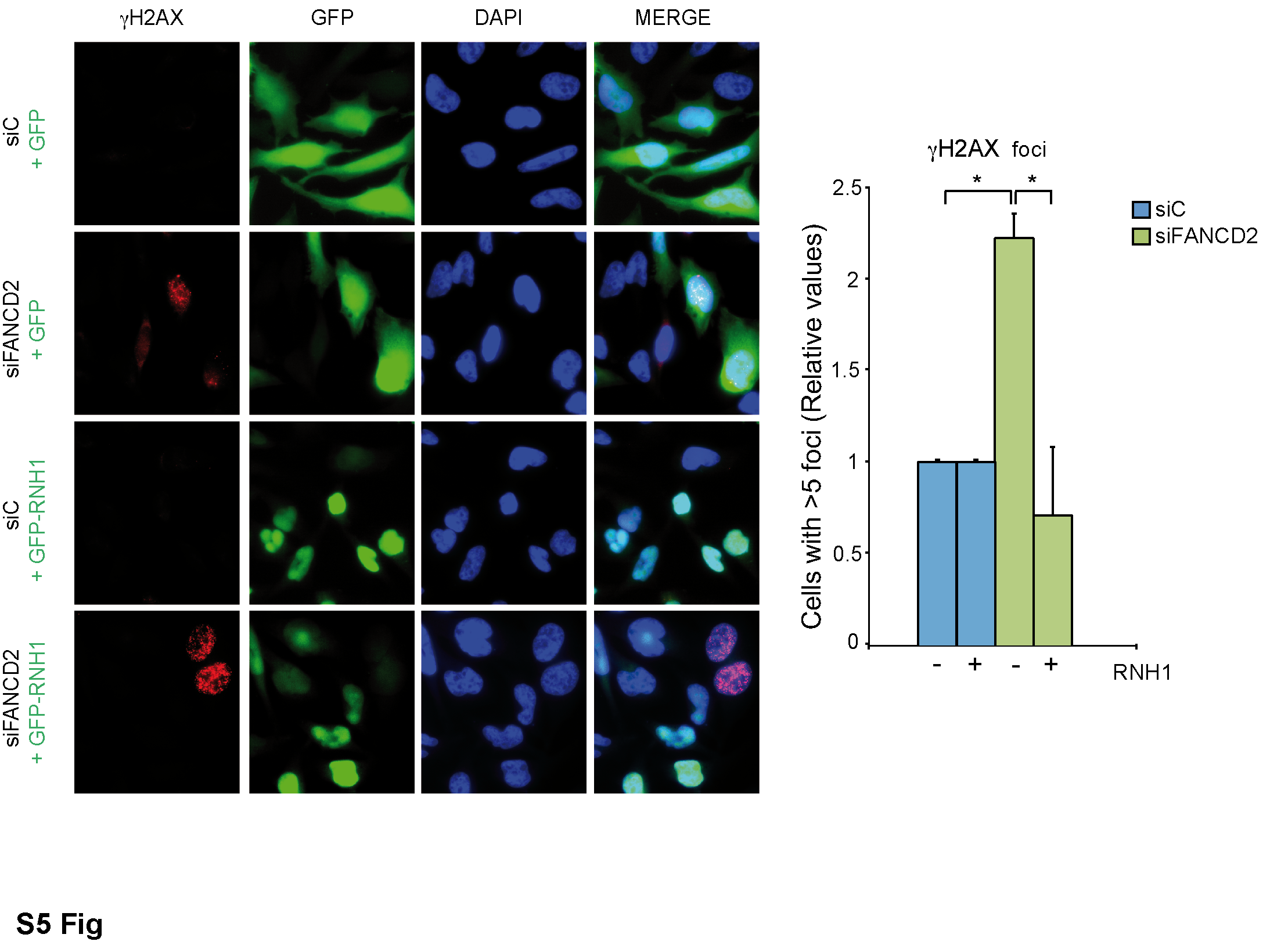

Supplement: S5 Fig — The graph shows the quantification of the relative amount of cells containing >5 foci with respect to the siC in each case. Data represent mean ± SEM from three independent experiments. *, P < 0.05 (Mann-Whitney U test). (TIF) [file pgen.1005674.s005.tif]

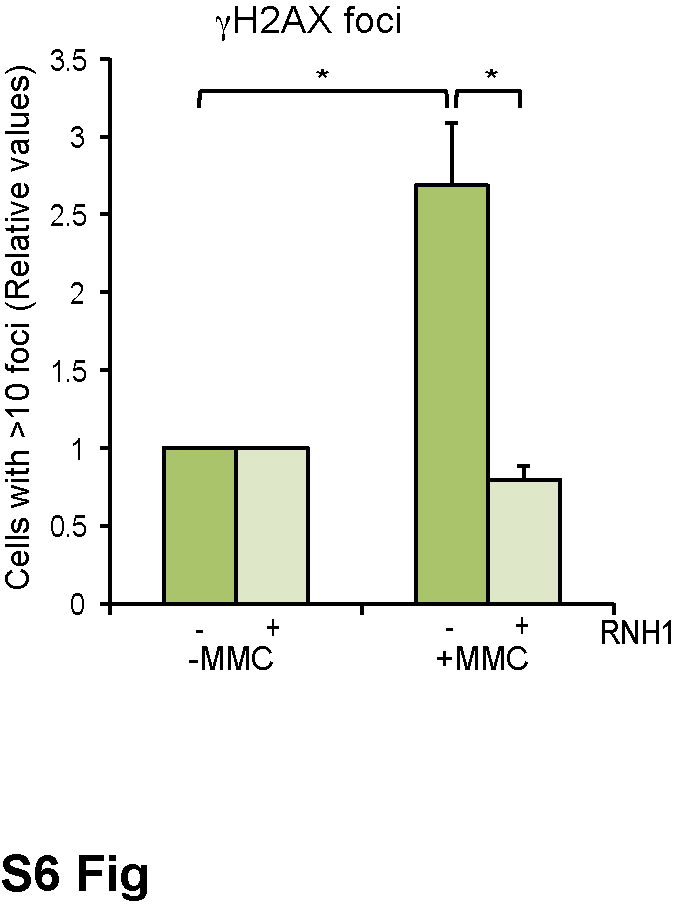

Supplement: S6 Fig — The graph shows the quantification of the relative amount of cells containing >10 foci with respect to the untreated (-MMC) cells. Data represent mean ± SEM from three independent experiments. * P < 0.05 (Mann-Whitney U test). (TIF) [file pgen.1005674.s006.tif]

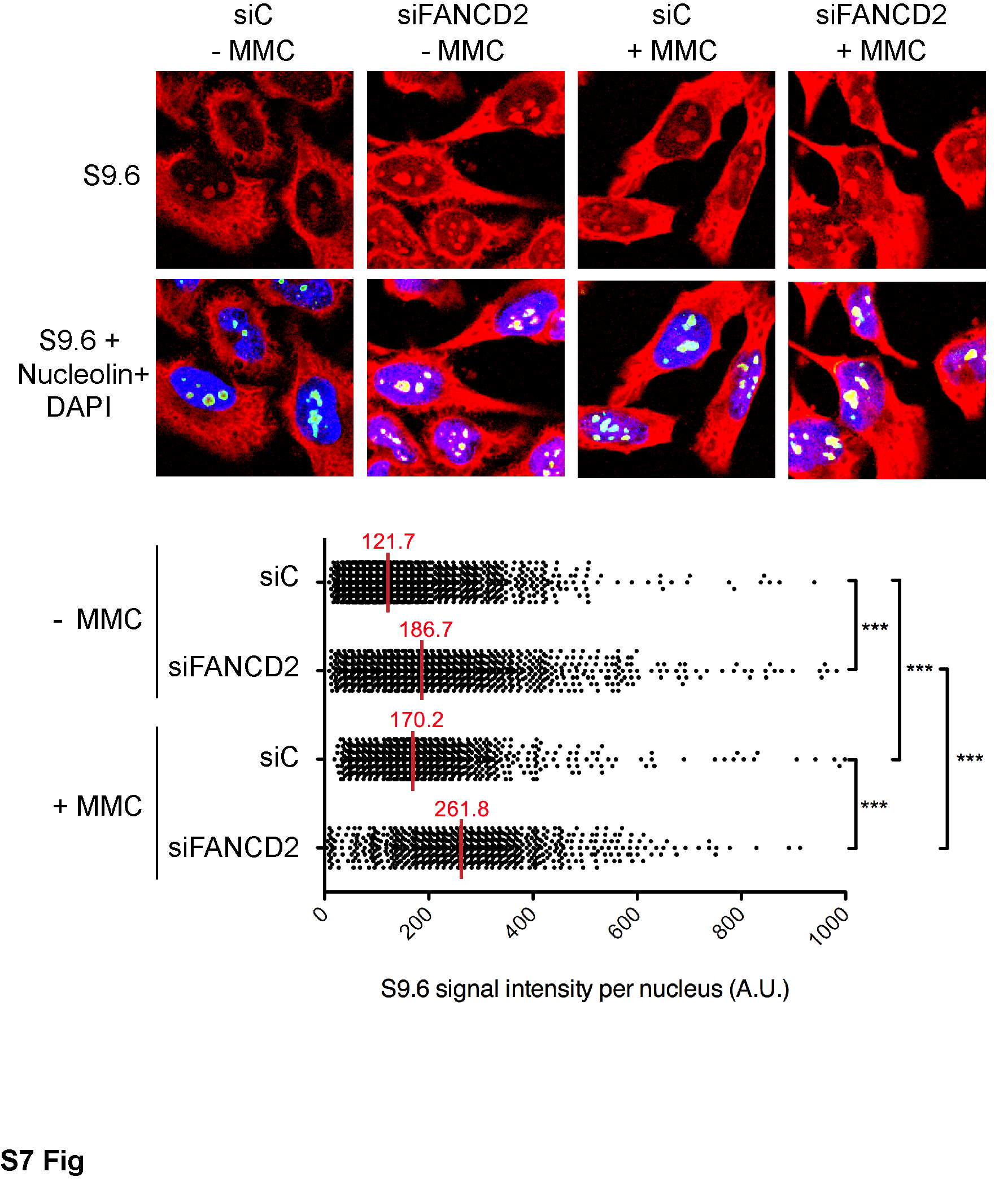

Supplement: S7 Fig — The graph shows the median of the S9.6 signal intensity per nucleus after subtraction of the nucleolar signal. More than 300 cells from four independent experiments were considered. ***, P < 0.001 (Mann-Whitney U test, two-tailed). (TIF) [file pgen.1005674.s007.tif]

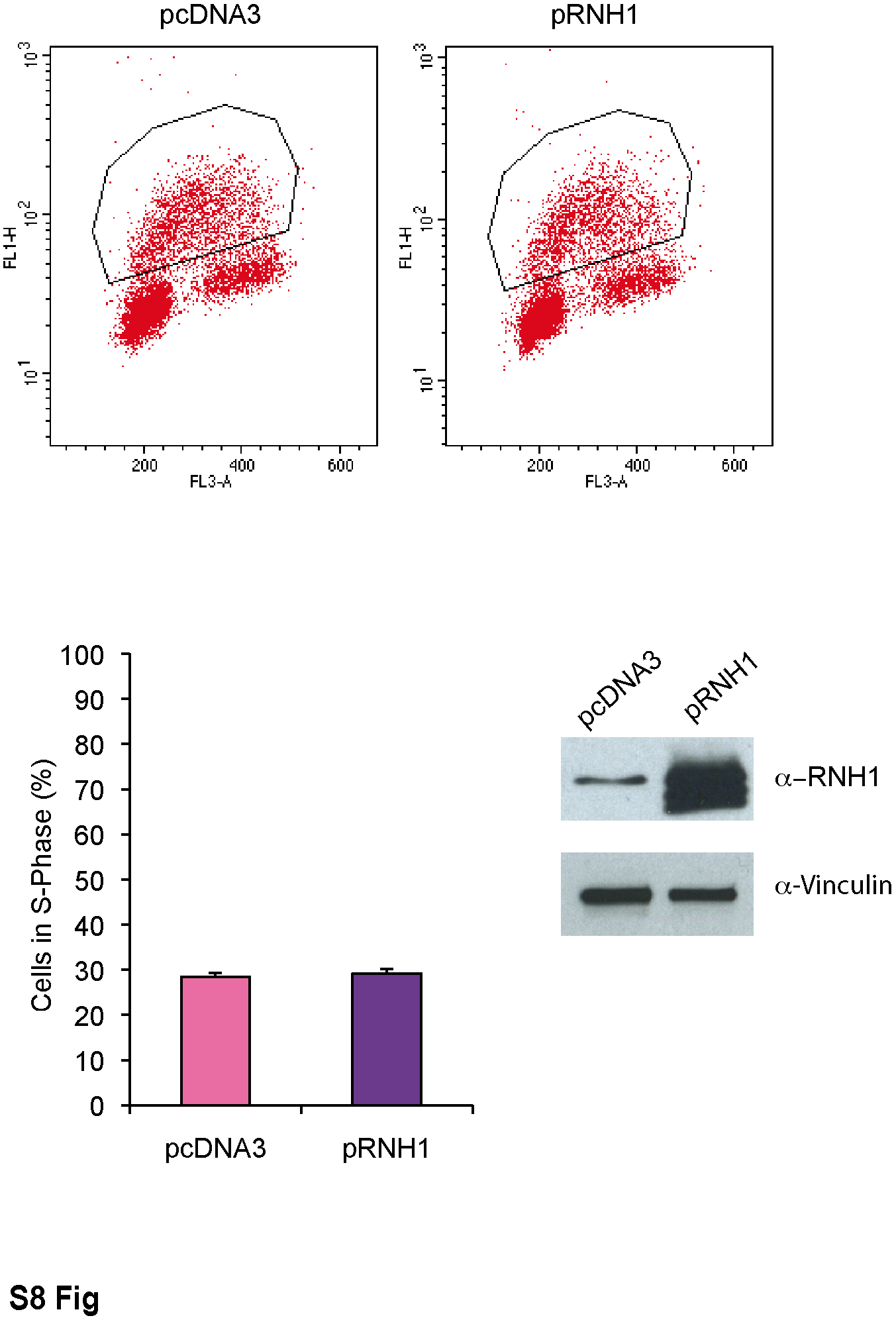

Supplement: S8 Fig — The graph shows the quantification of the percentage of cells in S phase. Data represent mean ± SEM from three independent experiments. Western blot shows the overexpression of RNase H1. The amount of Vinculin protein was used as a loading control. (TIF) [file pgen.1005674.s008.tif]
